# Development and evaluation of a novel capillary blood collection method for decentralized therapeutic drug monitoring using the True Dose kit

**DOI:** 10.1038/s41598-025-20951-5

**Published:** 2025-09-29

**Authors:** Nektarios Komninos, Serena De Chiara, Antonio Checa, Oscar Wiklander, Per Rydberg, Elham Hedayati

**Affiliations:** 1 True Dose AB, Stockholm, Sweden; 2 Unit of Integrative Metabolomics, Institute of Environmental Medicine, Karolinska Institute, 171 77 Stockholm, Sweden; 3 Unit for Biomolecular and Cellular Medicine, Department of Laboratory Medicine, Karolinska Institutet, Stockholm, Sweden; 4 Breast Center, Karolinska Institute, Comprehensive Cancer Center, Karolinska University Hospital, Stockholm, Sweden; 5 Department of Oncology-Pathology, Karolinska Institute, 171 77 Stockholm, Sweden; 6 Department of Oncology, South General Hospital, Stockholm, Sweden

**Keywords:** Capillary blood sampling, Anthracyclines, Epirubicin, Breast neoplasms/drug therapy, Blood specimen collection/methods, Mass spectrometry/methods, Cancer, Breast cancer, Cancer

## Abstract

Interindividual variability in epirubicin exposure limits the efficacy of standard chemotherapy dosing based on body surface area (BSA) in breast cancer. This study evaluated a novel liquid-based capillary microsampling technology, True Dose^®^, to enable decentralized therapeutic drug monitoring (TDM). It integrates internal standards (IS) at the blood collection point, enabling the immediate precipitation of proteins and stabilization of analytes. Analytical validation using liquid chromatography-tandem mass spectrometry (LC-MS/MS) showed that epirubicin signal responses from capillary samples corresponded with those from conventional venous samples, achieving R² values of ≥ 0.99. Intra-assay coefficient of variation (CV%) improved over time, decreasing from up to 18.6% at T0 to ≤ 11% from Day 3 onward, consistent with time-dependent matrix equilibration and improved extraction efficiency. Pre- and post-activation stability studies confirmed analyte integrity for up to 14 days at ambient temperatures. Hematocrit levels ranging from 7 to 18 g/dL showed no more than 17% signal variation, confirming matrix stability. In a clinical substudy involving 4 early-stage breast cancer patients, capillary-collected samples (*n* = 4 Cap-TD) yielded epirubicin/doxorubicin ratios that differed by no more than 15% from venous-derived samples; interpretation is limited by the small sample size. The findings support the feasibility of the True Dose^®^ system as a method for decentralized TDM of anthracycline (epirubicin) chemotherapy. The technology demonstrated analyte stability under room temperature storage for up to 14 days, time-dependent improvement in analytical precision, and minimal hematocrit bias, warranting further investigation in a broader clinical setting.

Clinical trial identification: EudraCT 2017-000641-44 (registered 27 Feb 2017); EUCT 2024-514818-12-00 (CTIS, transitioned from EudraCT).

**Supplementary Information:**

The online version contains supplementary material available at 10.1038/s41598-025-20951-5.

## Introduction

The variability in individual responses to chemotherapy underscores the critical need for enhanced methods of drug exposure measurement in oncology^1^. Standard dosing based on body surface area (BSA) frequently overlooks differences in drug metabolism and clearance, resulting in significant inter-individual variability in drug exposure (area under the curve (AUC))^2–4^.

Breast cancer remains the most common malignancy among women globally and is a significant public health concern^5,6^. In Sweden alone, over 8,800 women are diagnosed with breast cancer each year, highlighting the ongoing need to optimize treatment strategies to improve patient outcomes^7^. While advancements in diagnostics and therapies have significantly increased survival rates, substantial variability in treatment response persists, partly due to interindividual differences in drug exposure^8–11^. Therapeutic drug monitoring (TDM) offers a promising opportunity to personalize dosing and enhance treatment precision^12–16^. However, hospital-based TDM faces logistical barriers, including the need for venous access, immediate processing, cold-chain storage, and limited lab hours. These hinder timely sample collection at clinically optimal time points and compromise accurate drug exposure assessment^15,17–19^.

Capillary blood sampling, especially when combined with point-of-care stabilization technologies, offers a promising solution to the logistical challenges of Traditional TDM^20–22^. However, existing microsampling methods like dried blood spot (DBS) and volumetric absorptive microsampling (VAMS) face significant limitations, including hematocrit-dependent variability, inconsistent analyte recovery, complex sample pretreatment needs, and challenges maintaining sample stability^18,23,24^. To address these shortcomings, we evaluated the True Dose^®^ sampling kit. This novel capillary blood collection device incorporates internal standards (IS) and initiates immediate protein precipitation at the point of sample collection^25^. This design enables analyte stabilization without cold chain storage and supports decentralized, in-home TDM. By enhancing sample integrity and streamlining logistics, True Dose^®^ can potentially improve access to personalized chemotherapy monitoring and wider clinical implementation of TDM.

Chemotherapy remains a cornerstone in the treatment of early-stage breast cancer, particularly for women at elevated risk of recurrence^26,27^. Standard combination regimens, including anthracyclines (e.g., epirubicin) and taxanes, are used in approximately 40% of breast cancer cases, and they have been shown to reduce breast cancer–related mortality by about one-third compared to no chemotherapy^26^. However, the clinical benefit varies based on individual recurrence risk, and not all patients respond uniformly to standard dosing strategies. While novel cytotoxic agents have not yet surpassed the efficacy of anthracyclines and taxanes, therapeutic advancements have concentrated on improving dose intensity, administering more drug per unit of time, through dose-dense schedules and sequential administration^26^.

Despite advances in diagnostics and systemic therapies that have led to improved survival rates, the rising incidence of breast cancer and ongoing variability in treatment response underscore the necessity for optimized dosing. Variations in individual drug exposure can significantly impact therapeutic efficacy and toxicity. Therefore, developing and validating innovative and reliable methods for TDM is critical for advancing personalized medicine in oncology.

This proof-of-concept study aimed to evaluate the analytical performance and clinical feasibility of the True Dose^®^ capillary blood sampling kit compared to conventional venous blood collection for quantifying epirubicin by liquid chromatography-tandem mass spectrometry (LC-MS/MS). Using both spiked whole blood samples and clinical samples from four patients undergoing chemotherapy, we assessed the method’s accuracy, stability, and matrix effect tolerance, including hematocrit variability, as well as its real-world applicability. These preliminary findings support the potential of True Dose^®^ for advancing decentralized, personalized TDM in oncology.

## Materials and methods

### Reagents and materials

#### Chemicals and standards

Epirubicin-HCl (100 mg, Batch 3559), doxorubicin-HCl (150 mg, Batch D2975000), and daunorubicin-HCl (120 mg, Batch D0125000) were obtained from the British and European Pharmacopoeia Reference Standards. Organic solvents used to prepare the precipitation solution and the IS solvent composition are purchased from Sigma-Aldrich. Ultra-pure water was obtained using a Milli-Q water system (Merck Millipore).

#### Consumables and True Dose kit

True Dose^®^ capillary blood sampling devices (prototype versions 1.0–3.0) were supplied by True Dose AB (Solna, Sweden). Sample collection and processing used Sarstedt polypropylene microtubes (1.5 mL and 2.0 mL), prefilled with a standard precipitation solvent: isopropanol: methanol (1:1) containing 0.1% formic acid (IPA: MeOH, 1:1 with 0.1% formic acid). Sample homogenisation was facilitated using stainless steel beads and an Eppendorf^®^ Multipette^®^ E3.

### LC-MS/MS analysis

LC-MS/MS analysis was performed on a Waters Acquity Premier UPLC system (Binary Solvent Manager, Sample Manager, Column Manager) coupled to an Xevo TQ-S Triple Quadrupole Mass Spectrometer (Waters Corporation, Milford, MA, USA). Chromatographic separation was achieved using an ACQUITY UPLC HSS T3 column (2.1 × 100 mm, 1.8 μm, 100 Å) maintained at 35 °C, with an autosampler held at 8 °C.

Mobile phase A consisted of 0.1% formic acid in water, and mobile phase B consisted of 0.1% formic acid in acetonitrile. The flow rate was set at 300 µL/min. The chromatographic gradient employed was (time [min.], %B): 0.0, 25; 3.0, 45; 3.2, 72; 4.9, 72; 5.0, 100; 7.1, 100; 7.6, 25; 8.0, 25. A volume of 2 µL was injected for each sample.

Drugs were quantified by multiple reaction monitoring using electrospray ionisation in positive (ESI+). Quantifier and qualifier SRM transitions for epirubicin/doxorubicin were 544.3 → 397.0 and 544.3 → 379.0, respectively. Daunorubicin was employed as a secondary internal standard in the True Dose^®^ kit to cross-validate analytical signal stability. For daunorubicin, quantifier and qualifier transitions were 528.3 → 321.1 and 528.3 → 363.1, respectively. General MS parameters were set as follows: Capillary voltage of 3.0 kV, cone voltage of 25 V, desolvation temperature of 550 °C, desolvation gas flow of 600 L/h, and cone gas flow of 150 L/h.

Fresh calibration curves (7.8–1000 nM) were prepared on the day of analysis. Quality control (QC) samples at low, medium, and high concentrations were included with all test-runs for performance validation.

#### Calibration and quality control samples

Calibration curves and QC samples were prepared by spiking fresh human venous blood with known concentrations of epirubicin. The Lowest Limit of Quantification (LLOQ) was 4.1 nM, and the Upper Limit of Quantification (ULOQ) was 1000 nM. The calibration curve was prepared fresh on the day of analysis. Blood samples without added epirubicin (0 nM) served as blank controls. All QC samples were analyzed alongside study samples to monitor precision and accuracy across runs.

### Sample preparation development

True Dose^®^ product development and optimization occurred during 2024. Four successive versions (1.0, 2.0, 3.0, and 4.0) were evaluated throughout the year to enhance IS stability, mixing efficiency, and clinical evaluation. Table 1 outlines the key formulation and workflow differences between versions.

**Table 1:** Overview of True Dose kit versions and experimental Use; comparison of design features and specific experimental applications of True Dose kit versions 1.0 to 4.0 evaluated in 2024 for analytical equivalence, stability, and clinical validation studies.

Feature	1.0	2.0	3.0	4.0
Release Date	May 2024	October 2024	November 2024	December 2024
Matrix Type	PP version 1.0	PP version 2.0	PP version 3.0	PP version 3.0
Internal Standard (Conc.)	400 nM Dox/Dauno + additives	1 µM Dox/Dauno + additives	1 µM Dox/Dauno + additives	1 µM Dox/Dauno + additives
Microtube Volume	1.5 mL	1.5 mL	2.0 mL	2.0 mL
Precipitation Solvent Volume	500 µL	500 µL	1000 µL	1000 µL
Precipitation Solvent Composition	Organic solvents mixture	Organic solvents mixture	Organic solvents mixture	Organic solvents mixture
Steel Beads for mixing	Yes	Yes	Yes	Yes
Sample Volume (Blood)	20 or 50 µL	50 µL	50 µL	50 µL
Incubation time prior analytical work up	18 h, 3 days, 7 days, 14 days	3 days	3 days	3 days
Used for	Analytical equivalence, post-activation stability, hematocrit and initial clinical evaluation	Pre-activation stability and clinical evaluation	Pre-activation stability and clinical evaluation	Final analytical equivalence and pre/post-activation stability.

PP = polypropylene matrix; doxo/dauno = doxorubicin/daunorubicin.

### Traditional venous blood sample preparation for experimental use

For comparison, venous blood was collected into 10 mL EDTA tubes from healthy volunteers. In the lab, 10 µL of 1 µM doxorubicin (IS) and 500 µL of an organic precipitation solution (IPA: MeOH, 1:1 with 0.1% formic acid) were manually added. After vortexing for 30 s, the sample was either processed immediately (T0) or stored at − 26 °C for delayed analysis.

**Fig. 1:**
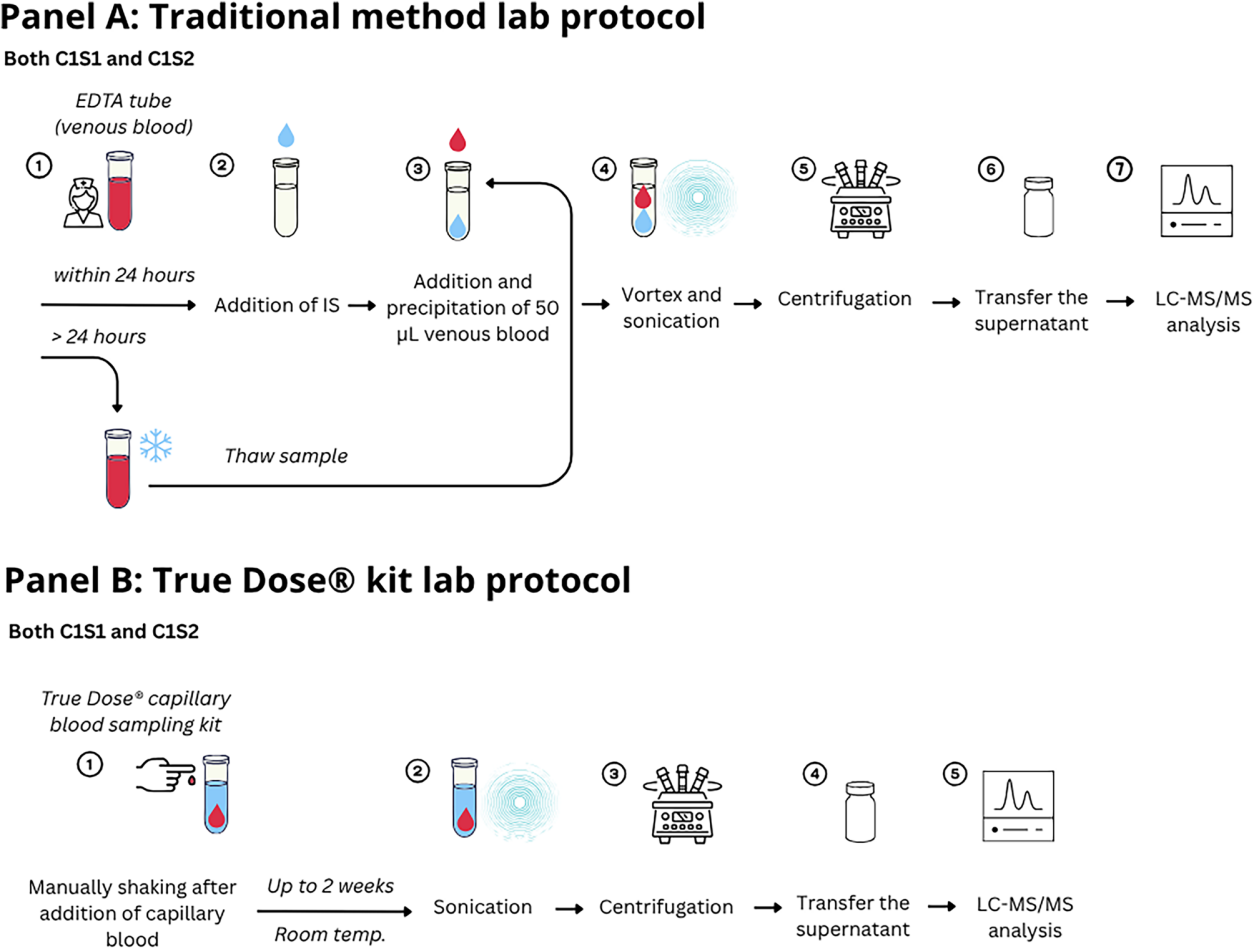
Comparison of traditional (**A**) and True Dose Kit (**B**) laboratory protocols for blood sample processing.

Figure 1 (Panel A) illustrates that the traditional workflow involved multiple steps, including manual IS addition, vortexing, thawing (if frozen), and subsequent sonication, centrifugation, and supernatant transfer.

### True Dose^®^ kit sample preparation for experimental use

Product versions 1.0 through 4.0 of the True Dose^®^ kit were developed and evaluated between May and December 2024. Each kit cap was preloaded with a liquid IS matrix and sealed. Upon activation, triggered by the addition of capillary blood, the matrix released the IS into the microtube’s extraction solvent. Depending on the version and study phase, either 20 µL or 50 µL of whole blood was added. The tube was then sealed and manually shaken for 30 s to ensure thorough mixing.

Following collection, samples were either analyzed immediately (T0) or stored at room temperature for 18 h, 3 days, 7 days, or 14 days before LC-MS/MS analysis. The laboratory preparation protocol for the True Dose^®^ kit is illustrated in Fig. 1 (Panel B). After the room-temperature incubation period, samples underwent sonication, centrifugation, and supernatant transfer for LC-MS/MS injection.

### Clinical sample collection and preparation (patient use)

For clinical validation, matched capillary and venous blood samples were obtained from patients at two time points following the epirubicin infusion. Each patient contributed one capillary sample collected using the True Dose^®^ kit (Cap-TD) and two 5 ml EDTA tubes of venous blood for reference and comparative analysis. Seven samples were prepared for each patient: one Cap-TD sample, three Lab-TD samples (venous blood processed using True Dose^®^ kits), and three Traditional samples (processed using the standard LC-MS/MS workflow).

Cap-TD (single): A nurse collected 20 or 50 µL of capillary blood using the True Dose^®^ kit. The sample was manually shaken for 30 s, stored at room temperature for 3 days, and placed on a thermomixer at 24 °C to simulate transport conditions.

Lab-TD (triplicates): 50 µL of venous blood was pipetted into activated True Dose^®^ kits. Samples were manually shaken for 30 s, stored at room temperature for 3 days, and placed on a thermomixer to simulate transport conditions.

Traditional (triplicates): Tubes were pre-filled with 1 ml of methanol and 20 µL of doxorubicin (IS) (1 µM in DMSO, without additives). Then, 50 µL of venous blood was added and vortexed for 30 s. Samples were immediately stored at − 26 °C for 3 days.

Post-Storage Processing (all sample types): On Day 3, all samples were vortexed for 10 s, sonicated in an ice bath for 10 min, and centrifuged at 9000 rpm for 10 min. A 150 µL aliquot of the supernatant was transferred to labelled amber insert vials and stored at − 26 °C until LC-MS/MS analysis.

## Experimental protocols

### Analytical equivalence study design

To assess the analytical equivalence between the True Dose^®^ sampling method and traditional venous blood collection, fresh venous blood was spiked with epirubicin at concentrations ranging from 0 to 1000 nM. These samples were aliquoted into both True Dose^®^ and traditional collection tubes. Additionally, True Dose^®^ samples were stored and analyzed after delayed intervals of 18 h, 3 days, 7 days, and 14 days to evaluate time-dependent performance. The comparability of the two sampling approaches was assessed by analyzing the signal ratios of epirubicin to IS (doxorubicin and daunorubicin), assessing regression slopes, R² values, and comparing the coefficient of variation (CV%) for each method and time point.

### Kit and sample stability: pre- and post-activation

#### Pre-activation stability study design

To evaluate the shelf life and matrix stability of the IS system in inactivated True Dose^®^ kits, a total of 38 kits were stored without blood at − 26 °C, 4 °C, and 25 °C for 7 and 14 days. After storage, kits were activated and processed using a blank extraction solvent to release the IS. The stability of doxorubicin and daunorubicin was assessed via LC-MS/MS by comparing AUCs with control samples from T0 (stored at − 26 °C). Stability was deemed acceptable if degradation stayed below 10% after 7 days and below 15% after 14 days. The doxorubicin-to-daunorubicin signal ratio was an additional internal control for assessing matrix consistency.

#### Post-activation stability study design

To assess the stability of epirubicin and IS after sample collection and activation of the True Dose^®^ kit, a total of 35 True Dose^®^ tubes containing blood were stored at room temperatures (25 °C) over a period of 14 days. LC-MS/MS was employed to evaluate analyte degradation by comparing AUC values at each time point with baseline samples. Stability was considered acceptable if degradation remained below 10% after 7 days and below 15% after 14 days.

### Clinical evaluation

#### Participant selection and study design

This clinical validation was embedded within the ongoing single-center TailorDose II study (EudraCT No. 2017-000641-44 and EUCT No. 2024-514818-12-00) at the Karolinska University Hospital Breast Centre. The trial is currently open for recruitment, and patients included in this feasibility phase were enrolled under ethical approval granted as part of the overarching TailorDose II study framework. The study protocol was approved by the Regional Ethics Review Board of the Karolinska Institute (Dnr 2019–02745 and 2024-02661-02), and all procedures adhered to the principles of the Declaration of Helsinki. Written and verbal informed consent was obtained from all participants prior to enrollment. Eligible participants included adults diagnosed with early-stage breast cancer who were scheduled to receive their first cycle of neoadjuvant or adjuvant epirubicin-based chemotherapy. Four patients were enrolled over two time periods: two from May to June 2024, and two from October to November 2024.

#### Sampling protocol

**Fig. 2:**
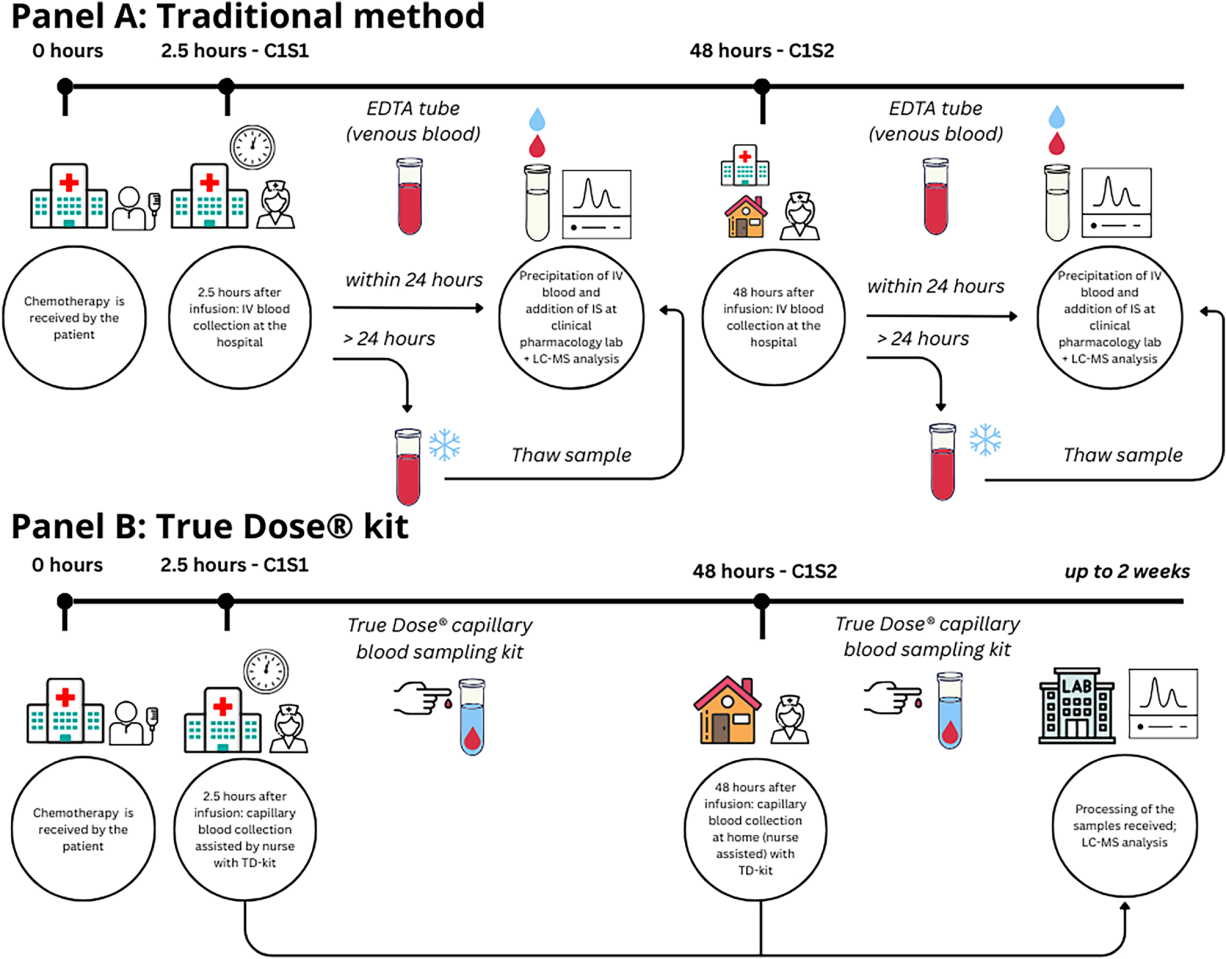
Workflow comparison between (**A**) traditional venous EDTA sampling and (**B**) capillary-based True Dose sampling for Cycle 1 Sample 1 (C1S1, 2.5 h) and Cycle 1 Sample 2 (C1S2, 48 h).

Each patient provided matched venous and capillary blood samples at two defined time points during the first chemotherapy cycle: Cycle 1, Sample 1 (C1S1): 2.5 h post-infusion, collected at the clinic; Cycle 1, Sample 2 (C1S2): 48 h post-infusion, collected at home by a mobile research nurse (Fig. 2A and B).

Two 5 mL venous EDTA tubes were collected and processed the same day using the standard LC-MS/MS workflow at each time point (Fig. 2A). In parallel, capillary samples (20 µL and 50 µL) were obtained using the True Dose^®^ capillary microsampling kit. These samples were activated, manually shaken for 30 s, and stored at room temperature for three days before analysis (Fig. 2B).

Both the 20 µL and 50 µL capillary formats were evaluated in May. Only the 50 µL format was utilized in the October-November cohort.

#### Capillary microsampling kit overview and collection process

**Fig. 3:**
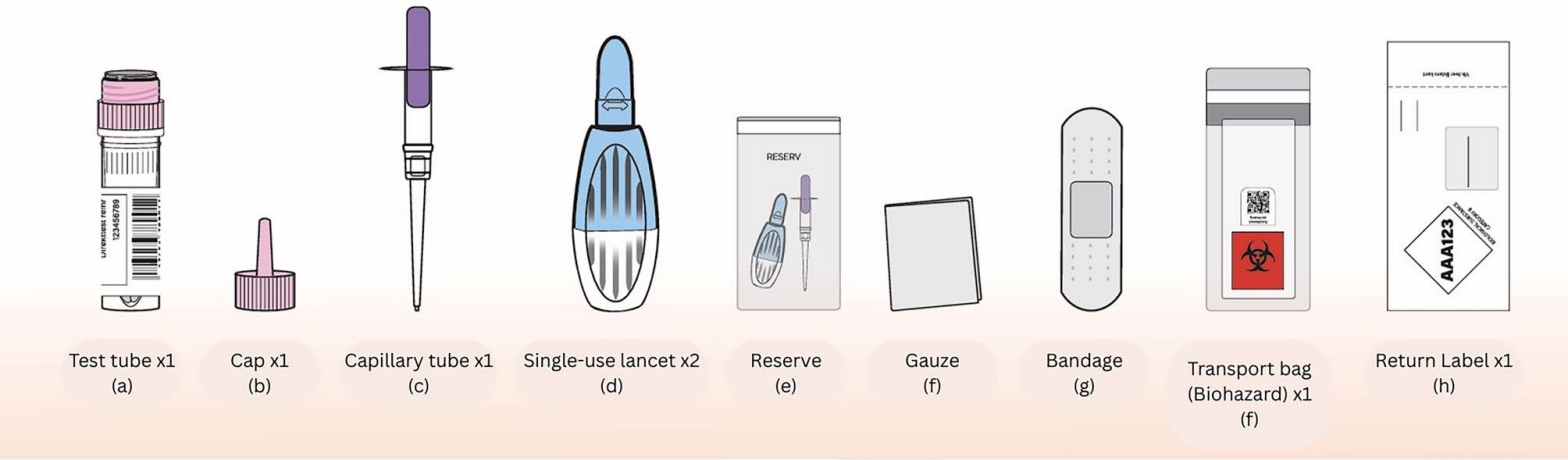
True Dose kit composition.

The True Dose^®^ kit included the following components: Instructions with QR-code-linked video guidance; a barcode-linked sample identification system; a BD Microtainer^®^ 2.0 mm safety lancet; a 50 µL Minivette^®^ POCT capillary tube; a 2 mL Sarstedt vial prefilled with 1000 µL of extraction solvent; supporting items: compress, plaster, biohazard bag, return label, and prepaid envelope (Fig. 3).

During sampling, patients were asked to wash their hands with warm water. A nurse assisted by pricking the fingertip with a lancet, collecting capillary blood using the Minivette^®^ pipette, and transferring it into a vial. The sample was then sealed, shaken vigorously for 30 s, and packed into a biohazard transport bag for return.

### Impact of hematocrit on analytical performance

Whole blood from a healthy individual was first centrifuged to separate red blood cells (RBCs) from plasma. These components were subsequently recombined in defined ratios to generate artificial blood samples with hematocrit concentrations ranging from 0 g/dL (plasma only) to 18 g/dL. A fixed concentration of epirubicin (37 nM) was spiked into each sample before reconstitution. The samples were then processed using the True Dose^®^ kit according to the standard protocol and analyzed via LC-MS/MS. AUC values for epirubicin were recorded and compared across various hematocrit levels to assess the impact of matrix composition on analytical recovery and signal response.

### Statistical analysis

All quantitative analyses were based on data generated by LC-MS/MS. The AUC for epirubicin was calculated using Waters TargetLynx™ software. Ratios between epirubicin and the IS doxorubicin and daunorubicin AUCs were also determined to evaluate analytical performance and comparability.

Several statistical parameters were used for all protocols conducted in this study, including the analytical equivalence study, pre-activation and post-activation stability tests, and the clinical sample evaluation. Linearity was assessed through linear regression analysis, and the calibration curves were required to meet a minimum R² threshold of 0.99. Analytical precision was expressed as the CV%, calculated using the formula: CV (%) = (Standard Deviation ÷ Mean AUC) × 100. A CV of no more than 11% was considered acceptable for all replicate LC-MS/MS measurements from day 3 onward.

Stability of both epirubicin and the IS was assessed by comparing AUC values across different storage durations. Degradation was deemed acceptable if it remained below 15% after 14 days of storage under the specified temperature conditions. The comparability of the True Dose^®^ and Traditional methods was assessed by examining differences in signal ratios (epirubicin/doxorubicin (epi/doxo)), as well as the alignment of regression slopes and R² values across various time points and sample types.

In the clinical setting, samples collected at 2.5 h and 48 h post-epirubicin infusion were compared to assess the accuracy and reproducibility of the True Dose^®^ method under real-world conditions. The comparison included inter-method agreement between capillary and venous blood samples, variability among replicates, and overall analytical consistency.

All statistical calculations were performed using Microsoft Excel.

## Results

The pre-analytical workflows differed between the True Dose^®^ capillary microsampling approach and the conventional venous reference method. The True Dose^®^ method required fewer manual steps, eliminating IS pipetting, vortexing, and thawing, while allowing for direct integration of the IS at the point of blood collection. Once activated, samples could be stored at room temperature for up to 14 days before analysis without compromising analyte integrity. In contrast, the Traditional method involved multiple manual interventions, including IS addition, vortexing, cold storage, and thawing before analysis. Figures 1 and 2 illustrate that the capillary-based method reduces operator-dependent handling steps. It eliminates the need for cold-chain storage, potentially streamlining the analytical process and improving sample throughput without compromising compatibility with LC-MS/MS analysis.

### Analytical equivalence: True Dose vs. traditional sampling

#### Dose–response comparison

Epirubicin dose-response curves (0-1000 nM) generated with the True Dose^®^ method demonstrated strong linearity across all measured time points. At T0, 35 Traditional and 34 True Dose^®^ samples (five replicates per concentration) were analyzed; one 37 nM replicate was omitted due to human error, and one 111 nM replicate with double the intended blood volume. For subsequent time points (18 h, 3 days, 7 days, 14 days), 15 samples were analyzed with five replicates each at 37 nM and 111 nM.

**Fig. 4:**
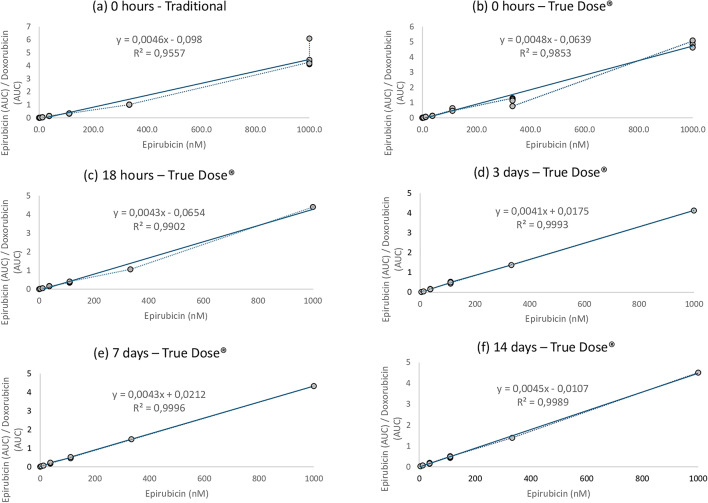
Linearity of Epirubicin Quantification Using Traditional vs. True Dose Sampling Over Time. Linear regression plots showing the ratio of Epirubicin (AUC) to Doxorubicin (internal standard) across increasing Epirubicin concentrations (0–1000 nM). Dose–response curves of epi/doxo ratios under six conditions: direct analysis (**a**) and True Dose at five time points (**b–f)**. Strong linearity (R² ≥ 0.99) was observed across 0–1000 nM Epirubicin, indicating analytical equivalence and stability.

At T0, linear regression yielded R² ≥ 0.95 for Traditional and R² ≥ 0.98 for True Dose^®^. At 18 h, R² increased to ≥ 0.99 for True Dose^®^. For later time points (3 d, 7 d, 14 d), R² was consistently ≥ 0.999 for True Dose^®^. The epi/doxo signal ratio between epirubicin (target drug) and doxorubicin (True Dose^®^ added IS), plotted against epirubicin concentration, displayed nearly identical slopes across methods and time points, supporting the analytical equivalence of the two approaches (Fig. 4). At T0, the Epi/Doxo ratio at 1000 nM agreed within 95.3% between Traditional and True Dose^®^ (Table 2), well within the ± 15% ICH M10 acceptance range^28^.

**Table 2:** Direct comparison of epirubicin and internal standard signal responses at baseline (T0) using traditional vs. True Dose methods.

Storage duration	Method	No. of samples (*n*)	Epi (AUC)	Dox (AUC)	Ratio epi/doxo (A.U.)	Daunorubicin (AUC)	Ratio epi/dauno (A.U.)	S/*N* (Epi)	% Agreement (Traditional/True Dose)	
T0	Traditional	35	48,312	10,639	4.63	NA	NA	3,240		
T0	True Dose	34	47,118	9,712	4.86	19,394	2,59	3,900	95.3	

AUC = area under the concentration-time curve; S/N = signal-to-noise ratio; epi/doxo = ratio of epirubicin to doxorubicin; epi/dauno = ratio of epirubicin to daunorubicin; NA = not applicable. All ratios are reported in arbitrary units (A.U.). % Agreement = (Traditional epi/doxo ÷ True Dose epi/doxo) × 100.

#### Signal ratios: epirubicin to internal standards

The analytical comparability of True Dose^®^ and Traditional venous sampling methods was further evaluated by assessing epirubicin signal ratios at 1000 nM relative to IS: doxorubicin and daunorubicin. Across five time points (T0, 18 h, 3 d, 7 d, and 14 d), Epi (AUC)/Doxo (AUC) ratios remained within a range of 95.3% to 112.1% when comparing True Dose^®^ to Traditional values, with a mean concordance of 103.7% (Tables 2 and 3). Epi/Dauno ratios ranged from 1.97 to 2.20, corresponding to 11.6% variation, indicating stable IS recovery and minimal drift (Table 3). This highlights the stability of IS recovery and negligible drift over time.

**Table 3:** Stability of epirubicin and internal standards in True Dose samples stored at room Temperature. Analysis of signal integrity and internal standard ratios at 18 h, 3 days, 7 days, and 14 days post-sampling using the True Dose kit.

Storage duration	Method	No. of samples (*n*)	Epirubicin (AUC)	Doxorubicin (AUC)	Ratio epi/doxo (A.U.)	Daunorubicin (AUC)	Ratio epi/dauno (A.U.)	S/*N* (Epi)	% Agreement (Traditional/True Dose)
18 h	True Dose	15	49,484	11,283	4.39	22,495	2.20	2,500	105.4
3 days	True Dose	15	51,612	12,496	4.13	26,113	1.98	4,400	112.1
7 days	True Dose	15	52,488	12,132	4.33	25,214	2.08	3,600	107.0
14 days	True Dose	15	42,101	9,316	4.52	21,398	1.97	4,200	102.4

AUC = area under the concentration–time curve; S/N = signal-to-noise ratio; epi/doxo = ratio of epirubicin to doxorubicin; epi/dauno = ratio of epirubicin to daunorubicin. All ratios are reported in arbitrary units (A.U.). % Agreement = (Traditional epi/doxo ÷ True Dose epi/doxo) × 100.

#### Epirubicin recovery over time

Epirubicin AUC increased with delayed processing, particularly at lower test concentrations. at 37 nM, rising to 147.1% of the T0 value by Day 3–7 and remaining > 125% at Day 14 (Table 4). This increase was also observed at 111 nM (up to 125.4% at Day 7), consistent with delayed matrix equilibration improving extraction efficiency.

**Table 4:** Post-Activation stability of epirubicin and internal standards in True Dose and traditional Samples. Descriptive summary of epirubicin, doxorubicin, and Daunorubicin responses across different time points and concentrations following blood addition.

Method-Time	Nr of replicates	Conc Epi (nM)	Mean Epi AUC	CV% (Epi AUC)	Mean Doxo AUC	CV% (Doxo AUC)	Epi/Doxo Ratio	CV% (Epi/Doxo)	Mean Dauno AUC	CV% (Dauno AUC)	Epirubicin AUC Relative to TD-T0 (%)
True Dose - T0	5	0.0	N/A	N/A	8,643	8.9	N/A	N/A	17,428	5.9	
	5	4.0	154	25.1	8,272	5.4	0.019	29.0	17,038	3.0	
	5	12.0	506	12.2	8,406	14.8	0.061	14.7	16,790	11.5	
	4*	37.0	1,313	9.0	9,740	10.2	0.135	2.6	20,431	16.4	100.0
	5**	111.0	4,547	8.1	8,636	20.0	0.538	13.6	17,754	17.7	100.0
	5	333.0	10,970	3.0	10,019	21.9	1.132	18.6	19,558	17.4	
	5	1000.0	47,118	2.5	9,712	4.7	4.858	4.5	19,354	8.2	
Traditional - T0	5	0.0	N/A	N/A	8,769	4.5	N/A	N/A	N/A	N/A	
	5	4.1	151	8.4	9,631	3.8	0.016	11.6	N/A	N/A	
	5	12.0	439	4.0	9,781	2.4	0.045	3.3	N/A	N/A	
	5	37.0	1,464	4.0	9,945	4.5	0.147	4.6	N/A	N/A	111.5
	5	111.0	3,398	4.2	10,376	6.2	0.328	5.0	N/A	N/A	74.7
	5	333.0	11,062	3.2	10,872	2.8	1.016	1.1	N/A	N/A	
	5	1000.0	48,312	2.7	10,639	13.1	4.630	18.0	N/A	N/A	
TD-18 h	5	37.0	1,313	3.9	9,721	8.4	0.136	11.6	20,035	8.0	100.0
	5	111.0	5,239	4.3	10,410	20.5	0.516	15.5	20,887	15.0	115.2
TD-3d	5	37.0	1,931	3.2	12,268	5.5	0.157	4.3	24,908	5.8	147.1
	5	111.0	5,690	5.0	11,427	3.1	0.498	7.1	22,912	3.7	125.1
TD-7d	5	37.0	1,930	6.5	9,994	9.2	0.193	11.0	23,416	10.2	147.0
	5	111.0	5,703	1.9	11,671	3.2	0.489	4.4	25,442	3.5	125.4
TD-14d	5	37.0	1,641	4.1	9,704	7.2	0.170	10.9	23,350	8.5	125.0
	5	111.0	4,725	3.6	9,903	5.4	0.478	7.2	24,122	5.1	103.9

AUC = Area under the concentration–time curve; CV% = Coefficient of Variation; Doxo = Doxorubicin; Dauno = Daunorubicin; Epi = Epirubicin; TD = True Dose; T0 = Direct.

Epirubicin AUC Relative to TD-T0 (%): Calculated as (Epirubicin AUC at each time point ÷ AUC at True Dose direct T0 for 37 nM or 111 nM) × 100. This value reflects the percentage of initial signal retained over time, used for post-activation stability assessment.

* One replicate was not analyzed due to human error.

** Double the intended blood volume was added to this replicate; analyte levels were adjusted by halving the measured values during analysis

#### Precision at low concentrations (< 37 nM)

At T0, replicate CV% for True Dose^®^ samples at < 37 nM exceeded the 10% precision threshold, ranging from 12.2 to 25.1% (Table 4). In contrast, Traditional sampling maintained CV% ≤ 8.4% in the same range. Data for < 37 nM were collected at later time points but not in replicate; therefore, time-dependent precision at these levels could not be evaluated (Supplemental Table 1).

#### Stability of epirubicin before and after sampling

##### Pre-activation matrix stability

True Dose^®^ kits that were stored in their inactivated (dry) state for 7 and 14 days at various temperatures (− 26 °C, 4 °C, and 25 °C) maintained robust matrix integrity. Analysis of doxorubicin and daunorubicin signals showed no more than 10% degradation at 7 days and below 15% at 14 days in all cases, which aligns with accepted bioanalytical stability criteria. Across all storage conditions, the CV% for the IS doxorubicin signal consistently remained below 7.5%. Additionally, the doxorubicin-to-daunorubicin ratio stayed stable within the range of 1.79 to 1.89, indicating no significant degradation or relative bias between standards. This confirms that the True Dose^®^ matrix preserves analyte integrity at room temperature and refrigerated for up to two weeks.

The control samples at time 0 (*n* = 6), stored at -26 °C, showed an average AUC of doxorubicin of 9410, a standard deviation (STDEV) of 385, and a CV of 4.09%. The average AUCs of doxorubicin at 14 days (*n* = 3) were 10,287, with a STDEV of 216 and a CV of 2.10%. The difference between time 0 and 14 days was 6.30%.

This experiment was conducted on version 4.0 in December 2024 (Table 1) to validate the data from previous studies.

##### Post-activation stability (blood added)

The stability of epirubicin and IS following blood addition was assessed over a 14-day period at 25 °C. The AUC degradation remained below 10% at 7 days and below 15% at 14 days. No significant signal loss was detected compared to T0 (Table 4). Across this time frame, CV% values for both epirubicin AUC and IS (doxorubicin AUC, daunorubicin AUC) decreased steadily (Table 4). Ratio values (Epi/Doxo and Epi/Dauno) also became more consistent, reflecting improved analyte-to-IS performance.

At 37 nM, Epi/Doxo CV% improved from 9.0% at T0 to 3.2% at Day 3, remaining ≤ 6.5% through Day 14. At 111 nM, CV% decreased from 13.6% at T0 and 15.5% at 18 h to 7.1% at Day 3, 4.4% at Day 7, and 7.2% at Day 14. IS showed the same stabilizing trend: doxorubicin CV% dropped from 20.0% at T0 to ≤ 5.5% from Day 3 onward, and daunorubicin CV% fell from 17.7% at T0 to ≤ 5.1% over the same period.

### Clinical evaluation

Four patients were enrolled in the study: two between May and June 2024, and two between October and November 2024. Two participants provided both capillary and venous blood samples at two time points: 2.5 h (Cycle 1 Sample 1, C1S1) and 48 h post-infusion (Cycle 1 Sample 2, C1S2), while two participants provided only C1S1 due to logistical issues with the mobile nurse. In total, six capillary blood samples were collected using the True Dose^®^ kit (Cap-TD) and six venous EDTA tubes were processed for Lab-TD and Traditional methods (each in triplicate).

### Capillary vs. venous True Dose and traditional sampling

**Fig. 5:**
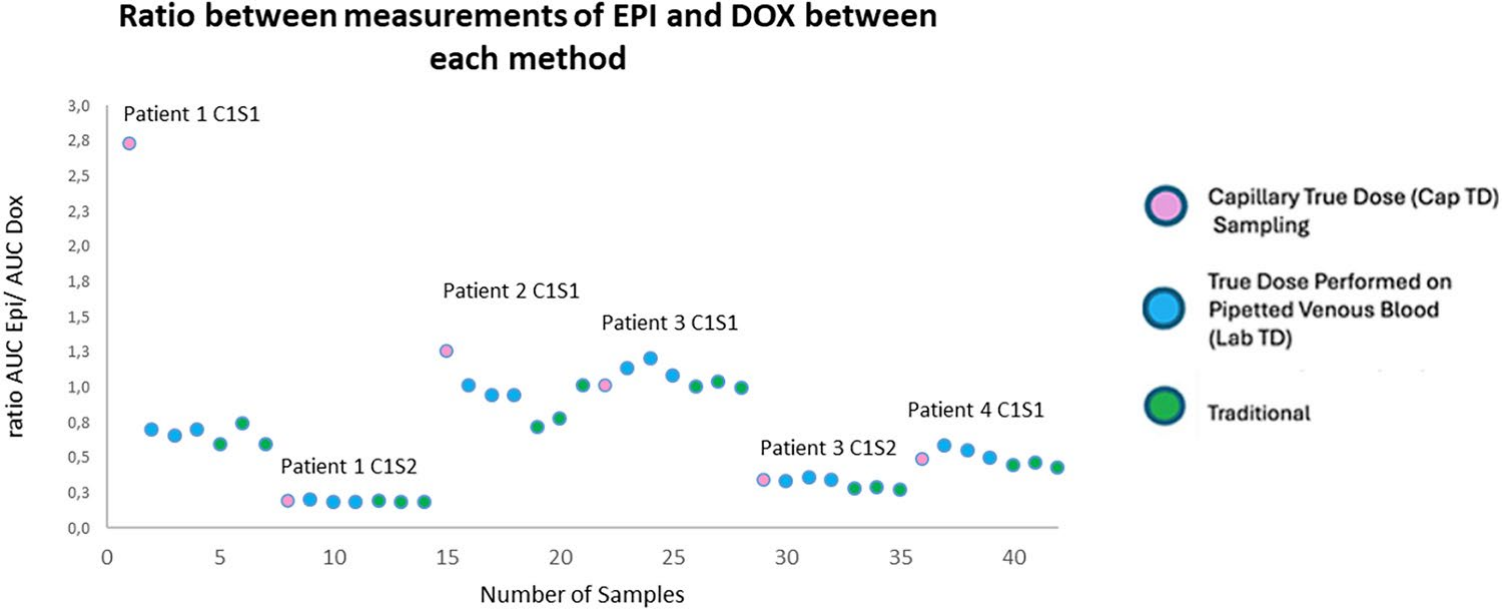
The ratio of epirubicin and doxorubicin between each different method.

Among the capillary patient samples (*n* = 4 Cap-TD) analyzed, analytical performance was compared across three methods: Cap-TD, Lab-TD, and Traditional; the epirubicin-to-doxorubicin (epi/doxo) signal ratios differed by less than ± 15%, demonstrating analytical agreement (Fig. 5).

Two Cap-TD samples from the first-generation kit (v1.0) were excluded from all comparative analyses due to internal standard degradation (Supplemental Table 3): one (P1 C1S1) with marked degradation (doxorubicin AUC = 1837) and one (P2 C1S1) with partial degradation (doxorubicin AUC = 5024). For the remaining Cap-TD samples (*n* = 4), each single capillary replicate was compared with triplicate Lab-TD venous replicates and triplicate Traditional venous replicates collected at the same time. Lab-TD replicates showed the lowest intra-method variability (CV% 3.9–8.5%), while Traditional replicates had the highest (up to 18.6%). Cap-TD results were generally equal to or lower in variability compared to both venous methods, and the epi/doxo ratio differed by ≤ 5% from the mean of the corresponding Lab-TD triplicates.

An exception was P3 (C1S1), where Traditional variability (2.0%) was lower than Cap-TD (5.2%), likely due to ion suppression at higher analyte levels (~ 800 nM). P4 (C1S1) showed slightly higher Cap-TD variability (8.8%) compared with Lab-TD (8.5%), which may reflect patient-specific sample composition. Overall, reproducibility was maintained across sampling types, and the Cap-TD and Lab-TD workflow demonstrated performance comparable to, or better than, Traditional venous sampling under most conditions (Table 5).

**Table 5:** Variability (CV%) of epi/doxo signal ratios in capillary (Cap-TD) and venous (Lab-TD) samples compared to traditional method, LC-MS/MS analysis.

Patient	Epirubicin Cycle	Sample	Cap-TD vs. Lab-TD CV%	Cap-TD vs. Traditional CV%	Intra-Lab-TD CV%	Intra-Traditional CV%	Notes
P1	C1	S1 (20 µL)	N/A*	N/A*	3.9	13.7	Higher variability in Traditional replicates, and low Doxo (AUC) in Cap-TD
P1	C1	S2 (20 µL)	4.4	3.3	5.1	3.2	Improved agreement at 48 h
P2	C1	S1 (20 µL)	N/A*	N/A*	4.1	18.6	Higher variability in Traditional samples, and low Doxo (AUC) in Cap-TD
P3	C1	S1 (50 µL)	7.0	1.6	5.2	2.0	Possible ion suppression at high concentrations (800 nM)
P3	C1	S2 (50 µL)	3.0	11.8	3.6	2.6	Traditional comparison yielded a higher CV%
P4	C1	S1 (50 µL)	8.8	6.0	8.5	3.9	Cap-TD showed slightly more variability

Cap-TD = Capillary blood processed using the True Dose kit; Lab-TD = Venous blood processed using the True Dose kit; Traditional = Standard LC-MS/MS method using venous blood; CV% = Coefficient of Variation; C1 = Cycle 1 of chemotherapy treatment; S1/S2 = Sample 1 (2.5 h post-infusion), Sample 2 (48 h post-infusion); Doxo = Doxorubicin; Epi = Epirubicin.

* Cap-TD samples were excluded from the comparison due to unexpected IS degradation.

### Effect of hematocrit on epirubicin quantification using the True Dose method

Blood samples with a spanning range of hematocrit levels (7–18 g/dL) were prepared to evaluate the influence of red blood cell content on epirubicin quantification using the True Dose^®^ method. All 12 samples (each analysed in duplicates) were spiked with 37 nM epirubicin and analysed by LC-MS/MS under standardized conditions (Table 6, Supplemental Table 2).

**Fig. 6:**
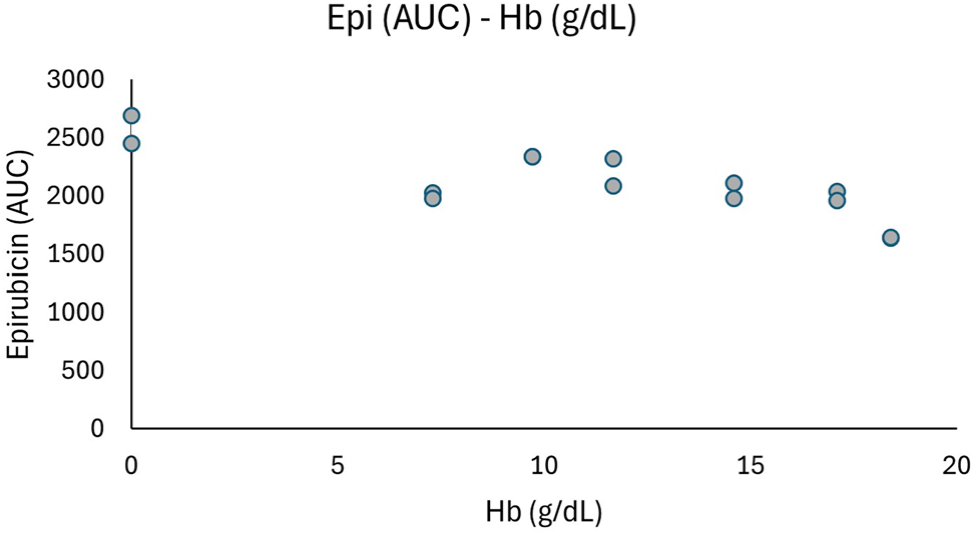
Impact of hematocrit levels (0–18 g/dL) on the AUC signal of epirubicin (37 nm) when analyzed with the True Dose method.

A weak inverse relationship between hematocrit levels and epirubicin signal (AUC) was observed, with a modest reduction in signal intensity at higher hematocrit concentrations. Across 7.3–18.4 g/dL, the mean AUC decreased by 18.2%. Inclusion of plasma (0 g/dL) indicated a total reduction of 36.4% from plasma controls to the highest hematocrit tested. A more pronounced decline was noted above 17 g/dL, indicating a modest matrix effect on analyte recovery at higher red blood cell content (Fig. 6).

**Table 6:** Measured hb (g/dL) values for different ratio of blood and plasma spiked with epirubicin (37 nM) and corresponding AUC values. Plasma-only samples (0 g/dL hb) serve as controls.

Haematocrit			
Version 1.0 TD	Description	Measured Hb values (g/dL)	Epirubicin (AUC)
Pure Plasma (EPI 37 μm)	Plasma	0	2,688
Pure Plasma (EPI 37 μm)	plasma	0	2,451
Blood: Plasma − 1:1	Hb 7	7.3	2,028
Blood: Plasma − 2:1	Hb 9	9.7	2,336
Blood: Plasma − 4:1	Hb 11	11.7	2,318
Pure Blood	Hb 14	14.6	2,109
Blood: Plasma − 1:1	Hb 7	7.3	1,979
Blood: Plasma − 2:1	Hb 9	9.7	2,340
Blood: Plasma − 4:1	Hb 11	11.7	2,085
Pure Blood	Hb 14	14.6	1,980
RBC: Blood − 1:5	Hb 16.8	17.1	2,038
RBC: Blood − 1:3	Hb 18.25	18.4	1,634
RBC: Blood − 1:5	Hb 16.8	17.1	1,960
RBC: Blood − 1:3	Hb 18.25	18.4	1,643

TD = True Dose^®^, Hb = Hemoglobin, AUC = Area under the concentration–time curve, RBC = Red Blood Cells.

## Discussion

This proof-of-concept study presents a novel liquid-based capillary microsampling technology, True Dose^®^, that integrates IS directly at the point of blood collection. This technology enables immediate protein precipitation and analyte stabilization, allowing robust performance in room temperature storage for up to 14 days. It is designed to overcome key limitations of traditional venous blood collection and DBS microsampling in TDM of chemotherapeutics. True Dose^®^ demonstrated analytical equivalence to venous sampling. Performance was confirmed in both spiked stability experiments and patient-derived clinical samples, with high linearity in spiked samples (R² ≥ 0.99), inter-method agreement in 4 patient samples tested (≤ 15% difference in epi/doxo ratios), and low intra-sample variability in patient venous triplicates (Lab-TD CV% 3.9–8.5%, meeting the ≤ 10% precision threshold). These findings address a major limitation in traditional workflows: sample degradation and incomplete extraction from blood matrices due to delayed processing or suboptimal storage conditions.

A distinctive finding was the time-dependent improvement in precision. In spiked True Dose^®^ replicates, intra-sample variability was highest at immediate processing (T0; CV% Epi/Doxo = 13.6% at 111 nM, 18.6% at 333 nM) but improved markedly with delayed workup, reaching ≤ 7.2% by Day 14. The same trend occurred in IS measurements (Dox CV%: 20.0% to ≤ 5.5%; Dauno CV%: 17.7% to ≤ 5.1% from Day 3 onward).

This stabilization likely results from a combination of protein-binding equilibration^29,30^, progressive red blood cell disintegration^31,32^, and improved solvent penetration into the intracellular compartment^33^. Such processes facilitate more complete drug release from erythrocytic and protein-bound compartments, leading to both higher recovery and lower variability over time.

These mechanistic benefits stem from the liquid-based design of True Dose^®^, which integrates the internal standard and protein-precipitating reagent directly within the sampling device. By eliminating plasma separation, drying, and manual IS addition, which can introduce variability in traditional venous or DBS workflows^20,25,33^, the kit produces pre-stabilized samples that are ready for direct LC-MS/MS analysis. This streamlined workflow avoids intermediate centrifugation or cold-chain transport, reducing technician workload, minimizing operator-dependent variability, and improving throughput, turnaround times, and overall reproducibility.

In spiked samples, our method achieved linearity (R² ≥ 0.999), low intra-method variability (CV% ≤ 11%) from day 3 onward, and signal integrity (> 95%) at room temperature. These metrics meet ICH M10 regulatory thresholds and exceed or match prior DBS and VAMS studies^18,20,23,28^. Meertens et al. validated hematocrit-independent VAMS for oral anticancer drugs, showing intra-assay CVs of 7–15%^18^. Additionally, Shafiei et al. reported up to 11% variability for capecitabine TDM via VAMS with minimal hematocrit interference^34^. Unlike these platforms, True Dose^®^ requires no hematocrit correction while maintaining reproducibility and precision across varied hematocrit levels under real-world ambient conditions. It also supports its adoption in oncology settings. These advantages align with recent trends summarized by Thangavelu et al., who highlighted the emergence of integrated microsampling systems to overcome traditional DBS and VAMS limitations, particularly regarding hematocrit bias, long-term stability, and clinical implementation feasibility^13,16,35^.

Although epirubicin AUCs at 37 nM and 111 nM increased at Day 3 and Day 7 (up to 147.1% and 125.4% of T0 values, respectively), this did not reflect a typical degradation trend. Instead, these elevations likely represent time-dependent changes in the sample matrix or extraction behavior. As these signals declined again by Day 14, the phenomenon appears transient. Across all post-activation timepoints, CV% for the epi/doxo ratio at 37 nM and 111 nM remained ≤ 11.0%, supporting stable relative quantitation over the 14-day period. In accordance with ICH M10 bioanalytical method validation guidelines^28^, long-term stability is deemed acceptable if the mean concentration deviates by no more than ± 15% from nominal values over the storage period. In our dataset, mean deviation from T0 was < 10% at Day 7 and within 15% at Day 14 for all analytes and concentrations tested, thereby meeting this regulatory threshold.

This preliminary stability evaluation was conducted over a wide concentration range, but not all concentrations were analyzed in replicates; thus, time-dependent precision improvement could only be confidently assessed at 37 nM and 111 nM. Ongoing studies will broaden the tested concentration range, assess longer storage durations (e.g., 21–30 days), and investigate potential analyte–matrix interactions to better understand the observed signal variability.

This early variability in True Dose^®^ likely reflects physical heterogeneity in the capillary blood/precipitation interface and matrix effects that are most pronounced during immediate processing. These trends confirm that the higher initial variability with True Dose^®^ is time-dependent, resolving as the matrix equilibrates. The improvement is consistent with known partitioning and precipitation kinetics: epirubicin is highly bound within red blood cells (~ 2:1 vs. plasma), and immediate precipitation at T0 can trap the drug within intact cells. Over 3–7 days, gradual cell disintegration, protein binding equilibration, and improved solvent penetration allow fuller extraction, reducing variability and increasing recovery.

The IS-stabilized capillary workflow is a significant advancement over DBS and VAMS, which often suffer from hematocrit bias, variable analyte recovery, and sample desiccation variability^23,24^. Our data showed a decrease of 18.2% in signal across hematocrit levels of 7.3–18.4 g/dL, while the greatest decline occurred above 17 g/dL, lower than the 30–40% hematocrit-related variation previously reported for other microsampling methods^24,36^. This is consistent with recent findings by Mazzarino et al., who showed minimal matrix interference when using liquid capillary blood for longitudinal hormone monitoring, supporting its utility in TDM workflows^37^. Hematocrit-dependent effects were assessed at 37 nM, a concentration representative of typical 48-hour post-infusion exposure in our clinical study. While future studies will include additional concentrations, 37 nM provided sufficient analytical sensitivity for this feasibility work and reflected real-world pharmacokinetic conditions.

In this proof-of-concept dataset from 4 patient samples, True Dose^®^ samples demonstrated low intra-method variability (Lab-TD CV% 3.9–8.5%) and close agreement (≤ 15% deviation in epi/doxo ratio) with venous samples. Although only one Cap-TD sample was available per time point, inter-method comparisons were possible by pairing each with triplicate Lab-TD samples from venous blood drawn in the same session (Cap-TD vs. Lab-TD CV% 4.4–8.8%, excluding 2 samples due to IS degradation).

The IS degradation observed in these two v1.0 Cap-TD samples likely resulted from a combination of human error during sealing, where even a slight leak could allow air exposure or partial solvent evaporation, and the lower initial IS concentration in v1.0 (400 nM vs. 1 µM in later versions), which made small losses appear proportionally larger in the AUC. Subsequent kit versions (≥ 2.0) incorporated design and procedural refinements, and no IS degradation was observed thereafter.

Variability in some patient sample results likely reflected biological rather than methodological factors. For example, P4 showed slightly higher Cap-TD variability, possibly due to differences in protein binding, endogenous metabolites, or concurrent medications. For P3 (C1S1), a high epirubicin concentration (~ 800 nM) near the upper clinical range may have caused minor ion suppression from co-eluting phospholipids and other endogenous components, rather than kit-derived additives.

These results align with earlier findings by Radovanovic et al. and Tripathy et al., who reported successful clinical application of patient-centric microsampling for paclitaxel and abrocitinib, respectively^21,36^. Notably, our findings extend this work by showing equivalence in an anthracycline-based cytotoxic chemotherapy setting, which has not been widely tested with existing microsampling kits. Furthermore, True Dose^®^’s internal IS integration at the point of collection minimizes operator-dependent variability, an improvement over previous self-collection studies^22^. This feature is crucial for ensuring data reproducibility and supports future unsupervised home-based TDM workflows. Harvey recently emphasized the importance of real-time pharmacokinetic data earlier in oncology care, underlining the translational potential of patient-centric microsampling for dose optimization^38^.

While Corsi et al. recently explored bioresorbable implantable sensors for doxorubicin TDM, highlighting advances in continuous monitoring technologies, these systems remain constrained by regulatory and technical barriers^39^. In contrast, True Dose^®^ offers a liquid-based, IS-preloaded microsampling solution with immediate translational applicability. From a clinical perspective, even in this small proof-of-concept set, the capillary method produced results closely aligned with matched venous samples. A similar patient-level dose-escalation strategy based on plasma exposure was recently reported in the OPTIMABI trial by Alexandre et al., reinforcing the clinical need for practical, scalable TDM tools in oncology^40^.

An important innovation in the True Dose^®^ capillary blood sampling kit is its enhanced stability profile during delayed processing. Samples stored at room temperature for 3 to 7 days retained over 85% of their initial signal and showed a reduced CV% compared to those samples that were processed immediately. This aligns with pharmacokinetic observations by El Azab et al., who noted improved epirubicin signal linearity with delayed extraction, possibly due to enhanced intracellular release or protein-bound analyte equilibrium^41^.

Traditional hospital-based TDM is constrained by sample timing, venous access, cold-chain transport, and lab availability^15,17,19^. These barriers often limit the feasibility of capturing optimal pharmacokinetic windows, such as 2.5–48 h post-infusion, critical for accurate dose personalization. Our successful collection of stable, analyzable samples at both clinical and home settings underpins the clinical flexibility of the True Dose^®^ system.

Our findings reinforce prior recommendations from Groenland et al. and Liu et al.^13^, emphasizing that real-time drug exposure monitoring is essential for optimizing dose intensity and minimizing toxicity. The reproducibility of the True Dose^®^ method, even at sub-therapeutic concentrations, provides a reliable basis for individualized chemotherapy adjustment in early breast cancer.

Study limitations include a small patient pool (*n* = 4), which precludes generalized pharmacokinetic inferences. Additionally, while nurse-assisted capillary sampling has been evaluated, fully unsupervised patient self-collection remains to be tested in real-world settings, an important step emphasized by Boffel et al. in adolescent capillary microsampling studies^22^. In the clinical comparisons, each time point relied on a single Cap-TD sample, limiting direct assessment of variability for capillary sampling. Furthermore, although stability was assessed across a broad concentration range, not all concentrations were run in replicates, which restricts statistical estimates at certain levels. Finally, two clinical samples (P1 C1S1 and P2 C1S1) processed with the first-generation kit (v1.0) were excluded due to IS degradation, which reduced the number of patient-derived data points and reflects design changes made during this proof-of-concept study.

These findings support further exploration of True Dose^®^ across additional drug classes and cancer types and potential integration with electronic health systems to enable real-time pharmacokinetic feedback in outpatient settings.

## Conclusions

In conclusion, this proof-of-concept study demonstrates that the True Dose^®^ system offers a hematocrit-insensitive and storage-stable alternative to traditional venous-based TDM, with preliminary evidence of pharmacokinetic equivalence and stability for up to 14 days at room temperature. Early variability at low concentrations (<35 nM) was limited to True Dose T0 processing, with stabilization achieved after 3 days.These findings support its potential as a platform for decentralized oncology care. Further validation in larger and more diverse patient cohorts, and across a wider range of drug classes, is warranted, while the findings in the clinical substudy should be interpreted with caution given the small clinical cohort (n=4).

## Supplementary Information

Below is the link to the electronic supplementary material.

Supplementary Material 1

Supplementary Material 2

Supplementary Material 3

## Data Availability

The datasets generated during the current study are not publicly available due to the privacy policy concerning private information of active army personnel. Full instrumental output reports are included under the “related” files section for access by the Editor and Reviewers during peer review. Still, they are available from the corresponding author upon reasonable request.
