# Supplementary material for: Development and evaluation of a novel capillary blood collection method for decentralized therapeutic drug monitoring using the True Dose kit
Source: Sci Rep. 2025 Sep 29;15:33331. doi: 10.1038/s41598-025-20951-5 (PMC12480760; doi:10.1038/s41598-025-20951-5)
Supplement: Supplementary file 1 — Supplementary Material 1 [file 41598_2025_20951_MOESM1_ESM.docx]

| **Supplemental Table 1.** Analysis of signal integrity and internal standard ratios at baseline (T0) using Traditional method, and at T0, 18 hours (18h), 3 days (3d), 7 days (7d), and 14 days (14 d) post-sampling using the True Dose kit. | | | | | | | | |
| --- | --- | --- | --- | --- | --- | --- | --- | --- |
| **Sample ID** | **1.Epi Conc Inc blood** | **2. Replicates** | **Stability ID** | **4. Epirubicin (AUC)** | **5. Doxorubicin (AUC)** | **6. Ratio Epi/Doxo(A.U.)** | **7. Daunorubicin (AUC)** | **8. Ratio Epi/Dauno(A.U.)** |
| **Traditional - T0** | | | | | | | | |
| Set1_001 | 0,0 | 5 | 1 | 0 | 8218 | N/A | N/A | N/A |
| Set1_002 | 0,0 |  | 2 | 0 | 8625 | N/A | N/A | N/A |
| Set1_003 | 0,0 |  | 3 | 0 | 9158 | N/A | N/A | N/A |
| Set1_004 | 0,0 |  | 4 | 0 | 9143 | N/A | N/A | N/A |
| Set1_005 | 0,0 |  | 5 | 0 | 8699 | N/A | N/A | N/A |
| Set1_006 | 4,1 | 5 | 6 | 166 | 9020 | 0,0184 | N/A | N/A |
| Set1_007 | 4,1 |  | 7 | 137 | 9686 | 0,0142 | N/A | N/A |
| Set1_008 | 4,1 |  | 8 | 162 | 9681 | 0,0167 | N/A | N/A |
| Set1_009 | 4,1 |  | 9 | 143 | 10004 | 0,0143 | N/A | N/A |
| Set1_010 | 4,1 |  | 10 | 145 | 9763 | 0,0148 | N/A | N/A |
| Set1_011 | 12,3 | 5 | 11 | 437 | 9938 | 0,044 | N/A | N/A |
| Set1_012 | 12,3 |  | 12 | 452 | 10058 | 0,0449 | N/A | N/A |
| Set1_013 | 12,3 |  | 13 | 462 | 9739 | 0,0474 | N/A | N/A |
| Set1_014 | 12,3 |  | 14 | 423 | 9739 | 0,0435 | N/A | N/A |
| Set1_015 | 12,3 |  | 15 | 423 | 9432 | 0,0448 | N/A | N/A |
| Set1_016 | 37,0 | 5 | 16 | 1401 | 10232 | 0,137 | N/A | N/A |
| Set1_017 | 37,0 |  | 17 | 1424 | 9609 | 0,148 | N/A | N/A |
| Set1_018 | 37,0 |  | 18 | 1485 | 10026 | 0,148 | N/A | N/A |
| Set1_019 | 37,0 |  | 19 | 1459 | 9375 | 0,156 | N/A | N/A |
| Set1_020 | 37,0 |  | 20 | 1551 | 10481 | 0,148 | N/A | N/A |
| Set1_021 | 111,1 | 5 | 21 | 3300 | 9318 | 0,354 | N/A | N/A |
| Set1_022 | 111,1 |  | 22 | 3310 | 10254 | 0,323 | N/A | N/A |
| Set1_023 | 111,1 |  | 23 | 3390 | 10644 | 0,318 | N/A | N/A |
| Set1_024 | 111,1 |  | 24 | 3344 | 10724 | 0,312 | N/A | N/A |
| Set1_025 | 111,1 |  | 25 | 3647 | 10938 | 0,333 | N/A | N/A |
| Set1_026 | 333,3 | 5 | 26 | 10894 | 10835 | 1,01 | N/A | N/A |
| Set1_027 | 333,3 |  | 27 | 11654 | 11264 | 1,03 | N/A | N/A |
| Set1_028 | 333,3 |  | 28 | 10932 | 10709 | 1,02 | N/A | N/A |
| Set1_029 | 333,3 |  | 29 | 11111 | 11075 | 1 | N/A | N/A |
| Set1_030 | 333,3 |  | 30 | 10718 | 10477 | 1,02 | N/A | N/A |
| Set1_031 | 1000,0 | 5 | 31 | 46652 | 10907 | 4,28 | N/A | N/A |
| Set1_032 | 1000,0 |  | 32 | 48870 | 10966 | 4,46 | N/A | N/A |
| Set1_033 | 1000,0 |  | 33 | 48317 | 11739 | 4,12 | N/A | N/A |
| Set1_034 | 1000,0 |  | 34 | 47629 | 11370 | 4,19 | N/A | N/A |
| Set1_035 | 1000,0 |  | 35 | 50092 | 8212 | 6,1 | N/A | N/A |
| **True Dose - T0** | | | | | | | | |
| Set1_036 | 0,0 | 5 | 36 | 0 | 9104 | N/A | 18371 | N/A |
| Set1_037 | 0,0 |  | 37 | 0 | 7698 | N/A | 16467 | N/A |
| Set1_038 | 0,0 |  | 38 | 0 | 8014 | N/A | 16322 | N/A |
| Set1_039 | 0,0 |  | 39 | 0 | 8833 | N/A | 17473 | N/A |
| Set1_040 | 0,0 |  | 40 | 0 | 9568 | N/A | 18505 | N/A |
| Set1_041 | 4,1 | 5 | 41 | 205 | 7812 | 0,0263 | 16806 | 0,0122 |
| Set1_042 | 4,1 |  | 42 | 185 | 8170 | 0,0227 | 16861 | 0,011 |
| Set1_043 | 4,1 |  | 43 | 115 | 8358 | 0,0138 | 17555 | 0,0066 |
| Set1_044 | 4,1 |  | 44 | 135 | 8036 | 0,0168 | 16403 | 0,0082 |
| Set1_045 | 4,1 |  | 45 | 131 | 8983 | 0,0145 | 17566 | 0,0075 |
| Set1_046 | 12,3 | 5 | 46 | 418 | 7501 | 0,0557 | 15753 | 0,0265 |
| Set1_047 | 12,3 |  | 47 | 511 | 7777 | 0,0657 | 15767 | 0,0324 |
| Set1_048 | 12,3 |  | 48 | 498 | 10461 | 0,0476 | 19928 | 0,025 |
| Set1_049 | 12,3 |  | 49 | 512 | 7582 | 0,0675 | 15192 | 0,0337 |
| Set1_050 | 12,3 |  | 50 | 592 | 8709 | 0,068 | 17311 | 0,0342 |
| Set1_052 | 37,0 | 4* | 52 | 1249 | 8911 | 0,14 | 17638 | 0,0708 |
| Set1_053 | 37,0 |  | 53 | 1490 | 11164 | 0,133 | 25272 | 0,059 |
| Set1_054 | 37,0 |  | 54 | 1275 | 9595 | 0,133 | 19700 | 0,0647 |
| Set1_055 | 37,0 |  | 55 | 1239 | 9289 | 0,133 | 19115 | 0,0648 |
| Set1_056 | 111,1 | 5 | 56 | 4924 | 9708 | 0,507 | 20745 | 0,237 |
| Set1_057 | 111,1 |  | 57 | 4765 | 9611 | 0,496 | 19186 | 0,248 |
| Set1_058 | 111,1 |  | 58 | 4304 | 7243 | 0,594 | 15183 | 0,283 |
| Set1_059 | 111,1 |  | 59 | 8058** | 12702** | 0,634 | 27291** | 0,295 |
| Set1_060 | 111,1 |  | 60 | 4713 | 10268 | 0,459 | 20008 | 0,236 |
| Set1_061 | 333,3 | 5 | 61 | 11397 | 8759 | 1,3 | 18141 | 0,628 |
| Set1_062 | 333,3 |  | 62 | 11071 | 9015 | 1,23 | 18548 | 0,597 |
| Set1_063 | 333,3 |  | 63 | 11108 | 9128 | 1,22 | 17079 | 0,65 |
| Set1_064 | 333,3 |  | 64 | 10579 | 9264 | 1,14 | 18462 | 0,573 |
| Set1_065 | 333,3 |  | 65 | 10697 | 13927 | 0,768 | 25561 | 0,418 |
| Set1_066 | 1000,0 | 5 | 66 | 48524 | 9583 | 5,06 | 18716 | 2,59 |
| Set1_067 | 1000,0 |  | 67 | 45220 | 9340 | 4,84 | 17613 | 2,57 |
| Set1_068 | 1000,0 |  | 68 | 47210 | 9262 | 5,1 | 18953 | 2,49 |
| Set1_069 | 1000,0 |  | 69 | 47164 | 10117 | 4,66 | 19595 | 2,41 |
| Set1_070 | 1000,0 |  | 70 | 47474 | 10258 | 4,63 | 21891 | 2,17 |
| **TD-18h** | | | | | | | | |
| Set1_086 | 0,0 | 1 | 86 | 0 | 10463 | N/A | 21753 | N/A |
| Set1_087 | 4,1 | 1 | 87 | 172 | 10050 | 0,0171 | 24252 | 0,0071 |
| Set1_088 | 12,3 | 1 | 88 | 485 | 9284 | 0,0522 | 19287 | 0,0251 |
| Set1_089 | 37,0 | 5 | 89 | 1362 | 8811 | 0,155 | 18517 | 0,0736 |
| Set1_090 | 37,0 |  | 90 | 1241 | 10665 | 0,116 | 22303 | 0,0556 |
| Set1_091 | 37,0 |  | 91 | 1301 | 9075 | 0,143 | 19246 | 0,0676 |
| Set1_092 | 37,0 |  | 92 | 1365 | 9611 | 0,142 | 19022 | 0,0718 |
| Set1_093 | 37,0 |  | 93 | 1298 | 10441 | 0,124 | 21089 | 0,0615 |
| Set1_094 | 111,1 | 5 | 94 | 5370 | 9975 | 0,538 | 20043 | 0,268 |
| Set1_095 | 111,1 |  | 95 | 5156 | 9831 | 0,525 | 20756 | 0,248 |
| Set1_096 | 111,1 |  | 96 | 4903 | 9508 | 0,516 | 19110 | 0,257 |
| Set1_097 | 111,1 |  | 97 | 5490 | 14103 | 0,389 | 26239 | 0,209 |
| Set1_098 | 111,1 |  | 98 | 5274 | 8633 | 0,611 | 18289 | 0,288 |
| Set1_099 | 333,3 | 1 | 99 | 12164 | 11254 | 1,08 | 23256 | 0,523 |
| Set1_100 | 1000,0 | 1 | 100 | 49484 | 11283 | 4,39 | 22495 | 2,2 |
| **TD-3d** | | | | | | | | |
| Set1_116 | 0,0 | 1 | 116 | 0 | 11800 | N/A | 23465 | N/A |
| Set1_117 | 4,1 | 1 | 117 | 195 | 11236 | 0,0174 | 22526 | 0,0087 |
| Set1_118 | 12,3 | 1 | 118 | 558 | 11756 | 0,0475 | 25130 | 0,0222 |
| Set1_119 | 37,0 | 5 | 119 | 1927 | 12499 | 0,154 | 24852 | 0,0775 |
| Set1_120 | 37,0 |  | 120 | 1929 | 12840 | 0,15 | 26813 | 0,0719 |
| Set1_121 | 37,0 |  | 121 | 1887 | 11245 | 0,168 | 22844 | 0,0826 |
| Set1_122 | 37,0 |  | 122 | 2033 | 12805 | 0,159 | 25471 | 0,0798 |
| Set1_123 | 37,0 |  | 123 | 1881 | 11950 | 0,157 | 24562 | 0,0766 |
| Set1_124 | 111,1 | 5 | 124 | 5735 | 11779 | 0,487 | 23314 | 0,246 |
| Set1_125 | 111,1 |  | 125 | 6030 | 11268 | 0,535 | 23364 | 0,2581 |
| Set1_126 | 111,1 |  | 126 | 5797 | 11080 | 0,523 | 22306 | 0,2599 |
| Set1_127 | 111,1 |  | 127 | 5251 | 11839 | 0,444 | 23802 | 0,2206 |
| Set1_128 | 111,1 |  | 128 | 5639 | 11169 | 0,505 | 21772 | 0,259 |
| Set1_129 | 333,3 | 1 | 129 | 16931 | 12234 | 1,38 | 23958 | 0,7067 |
| Set1_130 | 1000,0 | 1 | 130 | 51612 | 12496 | 4,13 | 26113 | 1,9765 |
| **TD-7d** | | | | | | | | |
| Set1_146 | 0,0 | 1 | 146 | 67*** | 10355 | 0,00649 | 24114 | 0,0028 |
| Set1_147 | 4,1 | 1 | 147 | 318 | 9696 | 0,0328 | 22272 | 0,0143 |
| Set1_148 | 12,3 | 1 | 148 | 672 | 10317 | 0,0652 | 25246 | 0,0266 |
| Set1_149 | 37,0 | 5 | 149 | 2069 | 11098 | 0,186 | 26674 | 0,0776 |
| Set1_150 | 37,0 |  | 150 | 1755 | 10593 | 0,166 | 24899 | 0,0705 |
| Set1_151 | 37,0 |  | 151 | 1869 | 9853 | 0,19 | 22870 | 0,0817 |
| Set1_152 | 37,0 |  | 152 | 2028 | 9729 | 0,208 | 21966 | 0,0923 |
| Set1_153 | 37,0 |  | 153 | 1928 | 8698 | 0,222 | 20673 | 0,0933 |
| Set1_154 | 111,1 | 5 | 154 | 5594 | 11446 | 0,489 | 25230 | 0,2217 |
| Set1_155 | 111,1 |  | 155 | 5731 | 11860 | 0,483 | 26074 | 0,2198 |
| Set1_156 | 111,1 |  | 156 | 5699 | 11525 | 0,494 | 24881 | 0,2291 |
| Set1_157 | 111,1 |  | 157 | 5626 | 12233 | 0,46 | 26594 | 0,2116 |
| Set1_158 | 111,1 |  | 158 | 5867 | 11290 | 0,52 | 24432 | 0,2401 |
| Set1_159 | 333,3 | 1 | 159 | 17859 | 12053 | 1,48 | 25605 | 0,6975 |
| Set1_160 | 1000,0 | 1 | 160 | 52488 | 12132 | 4,33 | 25214 | 2,0817 |
| **TD-14d** | | | | | | | | |
| Set1_176 | 0,0 | 1 | 176 | 157*** | 10586 | N/A | 23636 | N/A |
| Set1_177 | 4,1 | 1 | 177 | 273 | 10193 | 0,0267 | 23494 | 0,0116 |
| Set1_178 | 12,3 | 1 | 178 | 653 | 9716 | 0,0672 | 23781 | 0,0275 |
| Set1_179 | 37,0 | 5 | 179 | 1625 | 9807 | 0,166 | 23529 | 0,0691 |
| Set1_180 | 37,0 |  | 180 | 1562 | 10766 | 0,145 | 25749 | 0,0607 |
| Set1_181 | 37,0 |  | 181 | 1695 | 9037 | 0,188 | 21610 | 0,0784 |
| Set1_182 | 37,0 |  | 182 | 1724 | 9109 | 0,189 | 21122 | 0,0816 |
| Set1_183 | 37,0 |  | 183 | 1599 | 9801 | 0,163 | 24740 | 0,0646 |
| Set1_184 | 111,1 | 5 | 184 | 4742 | 9352 | 0,507 | 22974 | 0,2064 |
| Set1_185 | 111,1 |  | 185 | 4434 | 10304 | 0,43 | 25082 | 0,1768 |
| Set1_186 | 111,1 |  | 186 | 4842 | 9519 | 0,509 | 23519 | 0,2059 |
| Set1_187 | 111,1 |  | 187 | 4840 | 10620 | 0,456 | 25752 | 0,1879 |
| Set1_188 | 111,1 |  | 188 | 4768 | 9720 | 0,491 | 23282 | 0,2048 |
| Set1_189 | 333,3 | 1 | 189 | 14108 | 10147 | 1,39 | 26069 | 0,5412 |
| Set1_190 | 1000,0 | 1 | 190 | 42101 | 9316 | 4,52 | 21398 | 1,9675 |
| Abbreviations: AUC = Area under the concentration–time curve; Doxo = Doxorubicin; Dauno = Daunorubicin; Epi = Epirubicin; TD = True Dose; T0 = Direct.  * One replicate was not analyzed due to human error. ** Double the intended blood volume was added to this replicate; analyte levels were adjusted by halving the measured values during analysis  *** Sample was intended to be epirubicin-free (0.0 nM), but due to possible carryover from human error, a measurable concentration was detected. | | | | | | | | |
