# Supplementary material for: Development and evaluation of a novel capillary blood collection method for decentralized therapeutic drug monitoring using the True Dose kit
Source: Sci Rep. 2025 Sep 29;15:33331. doi: 10.1038/s41598-025-20951-5 (PMC12480760; doi:10.1038/s41598-025-20951-5)
Supplement: Supplementary file 2 — Supplementary Material 2 [file 41598_2025_20951_MOESM2_ESM.docx]

| **Supplemental Table 3.** Data values of Epirubicin, Doxorubicin, and Daunorubicin of Capillary (Cap-TD), Venous (Lab-TD), and the Traditional method. | | | | | | |
| --- | --- | --- | --- | --- | --- | --- |
| **Sample ID** | **Patient ID – sample type** | **Epi (AUC)** | **Dox (AUC)** | **Ratio Epi/Dox** | **Dauno (AUC)** | **Ratio Epi/Dauno** |
| Set1b_011 | Patient 1 (S1) Cap-TD | 5024 | 1837 | 2,7349 | 10685 | 0,470 |
| Set1b_012 | Patient 1 (S1) Lab-TD (1) | 5617 | 8050 | 0,698 | 16426 | 0,342 |
| Set1b_013 | Patient 1 (S1) Lab-TD (2) | 5033 | 7719 | 0,652 | 15297 | 0,329 |
| Set1b_014 | Patient 1 (S1) Lab-TD (3) | 5293 | 7575 | 0,699 | 16175 | 0,327 |
| Set1b_015 | Patient 1 (S1) Traditional (1) | 4363 | 7396 | 0,59 | N/A | N/A |
| Set1b_016 | Patient 1 (S1) Traditional (2) | 4295 | 5802 | 0,74 | N/A | N/A |
| Set1b_017 | Patient 1 (S1) Traditional (3) | 4487 | 7648 | 0,587 | N/A | N/A |
| Set1b_001 | Patient 1 (S2) Cap-TD | 1409 | 7432 | 0,190 | 15681 | 0,090 |
| Set1b_002 | Patient 1 (S2) Lab-TD (1) | 1348 | 6909 | 0,195 | 13307 | 0,101 |
| Set1b_003 | Patient 1 (S2) Lab-TD (2) | 1248 | 7042 | 0,177 | 13885 | 0,090 |
| Set1b_004 | Patient 1 (S2) Lab-TD (3) | 1157 | 6389 | 0,181 | 14401 | 0,080 |
| Set1b_005 | Patient 1 (S2) Traditional (1) | 1437 | 7602 | 0,189 | N/A | N/A |
| Set1b_006 | Patient 1 (S2) Traditional (2) | 1363 | 7641 | 0,178 | N/A | N/A |
| Set1b_007 | Patient 1 (S2) Traditional (3) | 1243 | 6925 | 0,18 | N/A | N/A |
| Set1b_031 | Patient 2 (S1) Cap-TD | 6323 | 5024 | 1,26 | 11386 | 0,555 |
| Set1b_035 | Patient 2 (S1) Lab-TD (1) | 6872 | 6793 | 1,01 | 16170 | 0,425 |
| Set1b_036 | Patient 2 (S1) Lab-TD (2) | 6582 | 6970 | 0,944 | 14568 | 0,452 |
| Set1b_037 | Patient 2 (S1) Lab-TD (3) | 6752 | 7182 | 0,94 | 14844 | 0,425 |
| Set1b_038 | Patient 2 (S1) Traditional (1) | 6495 | 9061 | 0,717 | N/A | N/A |
| Set1b_039 | Patient 2 (S1) Traditional (2) | 5516 | 7114 | 0,775 | N/A | N/A |
| Set1b_040 | Patient 2 (S1) Traditional (3) | 7850 | 7735 | 1,01 | N/A | N/A |
| Set2_027 | Patient 3 (S1) Cap-TD | 10581 | 10420 | 1,015 | 32798 | 0,323 |
| Set2_028 | Patient 3 (S1) Lab-TD (1) | 11862 | 10515 | 1,128 | 32277 | 0,367 |
| Set2_029 | Patient 3 (S1) Lab-TD (2) | 12471 | 10405 | 1,199 | 33102 | 0,377 |
| Set2_030 | Patient 3 (S1) Lab-TD (3) | 11813 | 10917 | 1,082 | 32682 | 0,361 |
| Set2_031 | Patient 3 (S1) Traditional (1) | 15841 | 15822 | 1,001 | N/A | N/A |
| Set2_032 | Patient 3 (S1) Traditional (2) | 15869 | 15377 | 1,032 | N/A | N/A |
| Set2_033 | Patient 3 (S1) Traditional (3) | 15602 | 15676 | 0,995 | N/A | N/A |
| Set2_040 | Patient 3 (S2) Cap-TD | 3112 | 9037 | 0,344 | 28741 | 0,108 |
| Set2_041 | Patient 3 (S2) Lab-TD (1) | 3417 | 10385 | 0,329 | 32114 | 0,106 |
| Set2_042 | Patient 3 (S2) Lab-TD (2) | 3824 | 10814 | 0,354 | 33944 | 0,113 |
| Set2_043 | Patient 3 (S2) Lab-TD (3) | 3376 | 9875 | 0,342 | 33427 | 0,101 |
| Set2_044 | Patient 3 (S2) Traditional (1) | 4275 | 15639 | 0,273 | N/A | N/A |
| Set2_045 | Patient 3 (S2) Traditional (2) | 4139 | 14545 | 0,285 | N/A | N/A |
| Set2_046 | Patient 3 (S2) Traditional (3) | 3670 | 13540 | 0,271 | N/A | N/A |
| Set2_053 | Patient 4 (S1) Cap-TD | 9245 | 18992 | 0,487 | 44545 | 0,208 |
| Set2_054 | Patient 4 (S1) Lab-TD (1) | 6163 | 10524 | 0,586 | 25878 | 0,238 |
| Set2_055 | Patient 4 (S1) Lab-TD (2) | 5521 | 10165 | 0,543 | 24974 | 0,221 |
| Set2_056 | Patient 4 (S1) Lab-TD (3) | 6247 | 12642 | 0,494 | 30529 | 0,205 |
| Set2_057 | Patient 4 (S1) Traditional (1) | 8492 | 19117 | 0,444 | N/A | N/A |
| Set2_058 | Patient 4 (S1) Traditional (2) | 7672 | 16828 | 0,456 | N/A | N/A |
| Set2_059 | Patient 4 (S1) Traditional (3) | 6017 | 14256 | 0,422 | N/A | N/A |
| Abbreviations: Epi = Epirubicin, Dox = Doxorubicin, Dauno = Daunorubicin, AUC = Area Under the concentration-time Curve, TD = True Dose, S1/S2 = Sample 1 (2.5h post-infusion), Sample 2 (48h post-infusion), Cap-TD = Capillary blood processed using the True Dose kit; Lab-TD = Venous blood processed using the True Dose kit; Traditional = Standard LC-MS/MS method using venous blood. | | | | | | |
