# Supplementary material for: Development and evaluation of a novel capillary blood collection method for decentralized therapeutic drug monitoring using the True Dose kit
Source: Sci Rep. 2025 Sep 29;15:33331. doi: 10.1038/s41598-025-20951-5 (PMC12480760; doi:10.1038/s41598-025-20951-5)
Supplement: Supplementary file 3 — Supplementary Material 3 [file 41598_2025_20951_MOESM3_ESM.docx]

| Supplemental Table 2. Measured Hb (g/dL) values for different ratios of blood and plasma spiked with epirubicin (37 nM) and corresponding AUC values. Plasma-only samples (0 g/dL Hb) serve as controls. | | | | |
| --- | --- | --- | --- | --- |
| Version 1.0 TD - Conditions | **Sample ID** | **Description** | **Measured Hb values (g/dL)** | **Epirubicin (AUC)** |
| Pure Plasma (EPI 37uM) | Set1_209 | Plasma | 0 | 2,688 |
| Pure Plasma (EPI 37uM) | Set1_210 | Plasma | 0 | 2,451 |
| Blood:Plasma - 1:1 | Set1_197 | Hb 7 | 7.3 | 2,028 |
| Blood:Plasma - 2:1 | Set1_198 | Hb 9 | 9.7 | 2,336 |
| Blood:Plasma - 4:1 | Set1_199 | Hb 11 | 11.7 | 2,318 |
| Pure Blood | Set1_200 | Hb 14 | 14.6 | 2,109 |
| Blood:Plasma - 1:1 | Set1_203 | Hb 7 | 7.3 | 1,979 |
| Blood:Plasma - 2:1 | Set1_204 | Hb 9 | 9.7 | 2,340 |
| Blood:Plasma - 4:1 | Set1_205 | Hb 11 | 11.7 | 2,085 |
| Pure Blood | Set1_206 | Hb 14 | 14.6 | 1,980 |
| RBC:Blood - 1:5 | Set1_201 | Hb 16.8 | 17.1 | 2,038 |
| RBC: Blood - 1:3 | Set1_202 | Hb 18.25 | 18.4 | 1,634 |
| RBC:Blood - 1:5 | Set1_207 | Hb 16.8 | 17.1 | 1,960 |
| RBC: Blood - 1:3 | Set1_208 | Hb 18.25 | 18.4 | 1,643 |
| Abbreviations: TD = True Dose, Hb = Hemoglobin, AUC = Area Under the Curve, RBC = Red Blood Cells. | | | | |
